# Differential miRNA expression profiles in proliferating or differentiated keratinocytes in response to gamma irradiation

**DOI:** 10.1186/1471-2164-14-184

**Published:** 2013-03-16

**Authors:** Nicolas Joly-Tonetti, José Viñuelas, Olivier Gandrillon, Jérôme Lamartine

**Affiliations:** 1 Université de Lyon, Université Claude Bernard Lyon I, Lyon, F-69003, France; 2 CNRS, UMR5534, Centre de Génétique et de Physiologie Moléculaires et Cellulaires, Villeurbanne, F-69622, France

**Keywords:** Primary keratinocytes, Epidermal differentiation, Gamma irradiation, miRNAs, Expression profiling

## Abstract

**Background:**

MicroRNAs (miRNAs), a group of short non-coding RNAs that negatively regulate gene expression, have recently emerged as potential modulators of cellular response to ionizing radiations both *in vitro* and *in vivo* in various cell types and tissues. However, in epidermal cells, the involvement of the miRNA machinery in the cellular response to ionizing radiations remains to be clarified. Indeed, understanding the mechanisms of cutaneous radiosensitivity is an important issue since skin is the most exposed organ to ionizing radiations and among the most sensitive.

**Results:**

We settled up an expression study of miRNAs in primary human skin keratinocytes using a microfluidic system of qPCR assay, which permits to assess the expression of almost 700 annotated miRNAs. The keratinocytes were cultured to a proliferative or a differentiated state mimicking basal or suprabasal layers of human epidermis. These cells were irradiated at 10 mGy or 6 Gy and RNA was extracted 3 hours after irradiation. We found that proliferative cells irradiated at 6 Gy display a global fall of miRNA expression whereas differentiated cells exposed to the same dose display a global increase of miRNAs expression. We identified twenty miRNAs weakly but significantly modulated after 6 Gy irradiation, whereas only 2 miRNAs were modulated after low-dose irradiation in proliferating cells. To go further into the biological meaning of this miRNA response, we over-expressed some of the responding miRNA in proliferating cells: we observed a significant decrease of cell viability 72 hours after irradiation. Functional annotation of their predicted targets revealed that G-protein related pathways might be regulated by these responding miRNAs.

**Conclusions:**

Our results reveal that human primary keratinocytes exposed to ionizing irradiation expressed a miRNA pattern strongly related to the differentiation status of irradiated cells. We also demonstrate that some miRNAs play a role in the radiation response to ensure the short-term survival of irradiated keratinocytes.

## Background

Ionizing radiations (IR) induce a large spectrum of damages that irradiated cells have to counteract by activating complex response pathways. The gene networks mobilized in the cellular response to radiations are subtly regulated. Several molecular actors have been shown to be involved in the regulation of gene expression after irradiation including chromatin-remodeling factors [1-3], transcription factors [4-6] or factors controlling protein synthesis [7], maturation [8] and degradation [9]. MicroRNAs (miRNAs) are short non-coding RNAs (21 to 23-nucleotide-long) which are predicted to control in mammals the activity of approximately 30% of all protein coding genes and have been shown to participate in the regulation of almost any cellular processes investigated so far [10]. By base pairing to the 3′ untranslated regions of target mRNAs, miRNAs mediate translational repression during the initiation or elongation step, and proteolysis of the nascent peptide or mRNA degradation after deadenylation [11].

miRNAs have been proposed to be important actors of the DNA damage response [12]. Several miRNAs expression studies have been conducted after irradiation in mice [13], human tumor samples [14], cancer cell lines [15,16] or normal human cells [15-18]. By crossing these studies, it was not possible to identify a short-list of universal miRNAs responding to IR. The radiation effect on miRNA expression seems therefore to vary according to cell type, radiation dose and post-irradiation time point [19].

Skin is the first organ to be targeted by external radiations and among the most sensitive. The epidermis is a stratified squamous epithelium that forms the protective covering of the skin. It is composed of a basal layer of proliferating cells, a spinous postmitotic cell layer, a granular layer, and a stratum corneum of terminally differentiating keratinocytes. High doses of radiation can induce various deleterious effects in skin, which can appear a short time after exposure, such as erythema and desquamation, and several years later, such as carcinoma and late complications [20]. Radiation-induced damages in epidermal proliferating cells have been suspected to be involved in late effects such as skin cancer whereas defects in differentiated cells could lead to early effects such as hyperkeratosis or desquamation. It is therefore of great interest to investigate the role of miRNAs in the radiation response in proliferating versus differentiated keratinocytes. Although the biological effects of low doses are controversial, we have previously shown that a very low dose of IR is able to induce a strong and specific mRNA response in human keratinocytes [21]. By providing a global view of the miRNA response after strong and low doses of IR, our study should help answering in which condition a miRNA response is induced after irradiation.

In this study, miRNA expression profiling was investigated in human primary keratinocytes after acute exposure to a very low (10 mGy) or a strong (6 Gy) dose of gamma rays. We showed that radiation induced changes in miRNA levels varied according to the dose and the differentiation status of irradiated keratinocytes. Indeed, after a strong dose of 6 Gy, a global repression of miRNAs was observed in proliferating keratinocytes whereas a global induction was detected in differentiated keratinocytes. The very low dose of 10 mGy was also able to modulate some miRNAs in proliferating cells. Over-expression of the most stringently regulated miRNAs in proliferating cells modified their survival after 6 Gy irradiation. In differentiated keratinocytes, repression of the 3 most activated miRNAs had no detectable effect on survival. Our data suggest that the miRNA response is globally modulated in irradiated keratinocytes and could be involved in cellular pathways that are highly dependent to the differentiation state of the IR-exposed cells.

## Results

### Characterization of the cellular model

**Figure 1:**
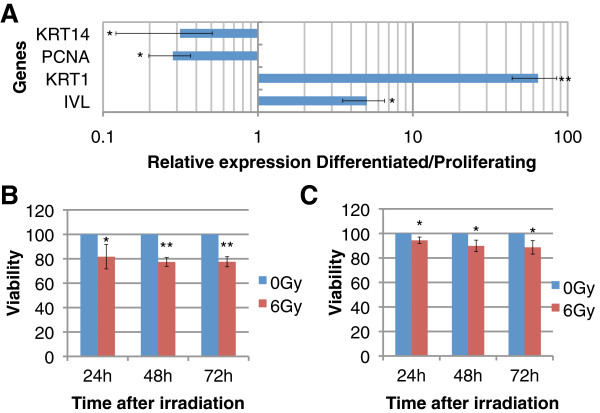
**Characterization of the cellular model.** (**A**) RT-qPCR analysis of epidermal basal cells markers (PCNA, KRT14) and suprabasal cells markers (IVL, KRT1) between proliferating and 3-days differentiated keratinocytes (n=3; error bars represent SD; bilateral paired t-test: *p≤0.05, **p≤0.01). (**B**) Viability assay between 6Gy-irradiated and sham-irradiated proliferating keratinocytes. Results are expressed as the mean percentage of viable cells assuming 100% viability for non-irradiated cells (n=3; error bars represent SD; bilateral paired t-test : *p≤0.05, **p≤0.01, ***p≤0.001). (**C**) Viability assay between 6Gy-irradiated and sham-irradiated differentiated keratinocytes. Results are expressed as the mean percentage +/− SD of viable cells of 4 independent experiments assuming 100% viability for non-irradiated cells (bilateral paired t-test: *p≤0.05).

The cellular model used in this study consisted of primary cultures of human keratinocytes (HPK) isolated from infant foreskin. For modeling epidermal basal cells, second passage cultures were cultivated up to 50% of confluency where most of the cells are still proliferating. To induce epidermal differentiation, cells were cultured at low calcium concentration up to confluence, and then incubated 3 days post-confluence in a culture medium containing 1.8 mM of calcium. Differentiated cultures were subjected to expression analysis by real-time PCR of two well-known markers of epidermal suprabasal layers: keratin 1 (KRT1) and involucrin (IVL) as well as markers of proliferating basal cells: keratin 14 (KRT14) and PCNA. The expression was compared to that of proliferative cultures. As expected, a strong expression of KRT1 and IVL transcripts was observed in differentiated keratinocytes whereas PCNA and KRT14 were clearly repressed (Figure 1A). These results suggest that the majority of cells cultured 3 days post-confluence in a high-calcium medium exhibited a differentiation state close to that found in spinous and granular layers. On the contrary, semi-confluent keratinocytes express markers typical to the basal layers of the human epidermis. We next investigated the short-term radiosensitivity of proliferating and differentiated keratinocytes after the acute high dose of 6 Gy. The viability of irradiated cells was measured 24 to 72 hours after irradiation (Figures 1B and 1C). For proliferating cells (Figure 1B), we observed a significant decrease in cell viability starting 24 h post irradiation (Student *t*-test, *P* < 0.05) with a maximal effect intensity of 20% decrease 48 h and 72 h post-irradiation (Student *t*-test, *P* < 10^-2^). In differentiated cells (Figure 1C), a weaker but significant effect was observed from 24 h to 72 h post-irradiation with a maximal decrease in cell viability of 10% 3 days post-irradiation (Student *t*-test, *P* < 0.05). These data confirmed, as previously reported by our group [21], that differentiated keratinocytes are more radio-resistant than proliferative keratinocytes.

### MiRNA expression profiling

We used TaqMan quantitative real-time PCR Low Density Array (TLDA) to profile miRNAs expression in control and irradiated keratinocytes. We investigated the miRNA response to a high dose of 6 Gy in proliferating and differentiated keratinocytes and to a very low dose of 10 mGy in proliferating cells, which are more radiosensitive. MiRNAs expression was assessed 3 hours post-irradiation since this time-point corresponds to the immediate gene response in irradiated keratinocytes [22] and miRNAs might be involved in the regulation of this gene response. Three to four human keratinocytes cultures established from independent donors were analyzed at each dose. Due to the high repeatability of the TLDA methodology [23,24], we privileged in our experimental plan biological replicates from different donors than technical repeats. It is important to note that the experimental plan is constructed on paired data: for each donor, a reference condition response and a response following ionizing irradiation were quantified.

**Figure 2:**
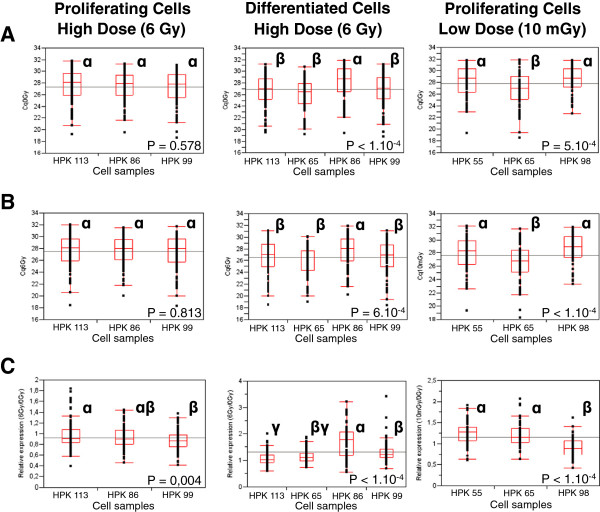
**Global analysis of miRNA-Cq distribution and relative expression after irradiation in the different HPK samples (All-paired Tukey-Kramer tests).** Each point represents a miRNA Cq in non-irradiated (**A**) or irradiated (**B**) state, or miRNA relative expression (irradiated vs non-irradiated) (**C**). Box & whiskers plots represent first decile, first quartile, medium, third quartile and ninths decile of data in each cell samples. The black line corresponds to the mean Cq for all samples. Greek letters besides box plots indicate differences between means: same letters-donors indicate no difference between means, different letters-donors indicate significant difference of means, double letters-donors indicate no difference with single letters.

Out of 667 miRNAs studied, only those with a Cq (quantification Cycle) value below 32 cycles in all samples were considered as significantly expressed. This threshold was calculated using miRNA duplicate and snRNA (RNU6-1, SNORD44, SNORD48) tetraplicate repeatability, in agreement with literature [25]. 95 miRNAs for 0 Gy vs 6 Gy (0/6 Gy) irradiated proliferating keratinocytes, 100 miRNAs for 0/6 Gy-irradiated differentiated keratinocytes, and 64 miRNAs for 0 Gy vs 10 mGy (0/10 mGy) irradiated proliferating keratinocytes were therefore considered for further analysis (see Additional File 1 for a complete list of these miRNAs and their corresponding Cq in the various conditions). We first investigated if a bias exists in the average expression of miRNAs between HPK cell samples sorted by differentiation state and irradiation dose (Figure 2). We used all-paired Tukey-Kramer tests to compare Cq means within HPK samples. For 0/6 Gy-irradiated proliferating keratinocytes, there was no expression bias between the cell samples (ANOVA *F*-test, p-value = 0.578 and 0.813 respectively). For 0/6 Gy-irradiated differentiated keratinocytes, significant differences were observed: samples from HPK86 revealed higher Cq than the three others donors (ANOVA *F*-test, *P* < 10^-4^ and 6.10^-4^ respectively). A difference was also observed for 0/10 mGy-irradiated proliferating keratinocytes: samples from donor HPK65 showed in average lower Cq, than those from HPK55 and HPK98 (ANOVA *F*-test, *P* = 5.10^-4^ and < 10^-4^ respectively). However, these differences in the average expression of miRNAs were similar for reference and irradiated conditions (compare panels A to panels B), and could be due to inter-individual variability in primary cells. This observation underlined the importance to use paired data for statistical analyses. It further confirmed that there was no sample processing bias due to irradiation state and that raw data normalization was not necessary as previously proposed [26].

**Figure 3:**
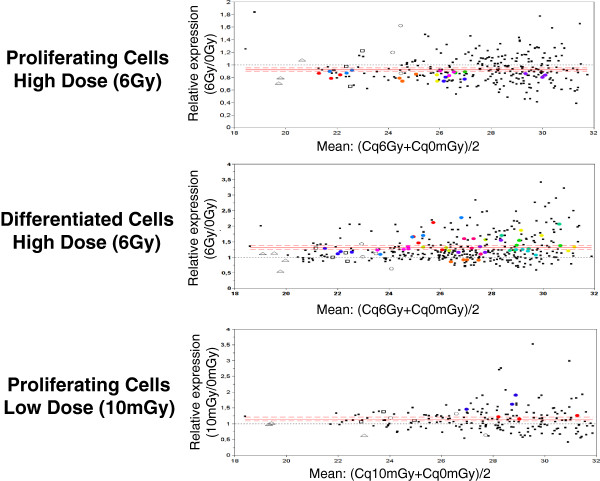
**Relative miRNA expression profiling for all cell samples.** Each dot corresponds to one miRNA analyzed in one cell sample. The mean Cq is plotted on the *X* axis whereas the relative expression (2ΔΔCq) is plotted on the *Y* axis. Continuous and discontinuous red lines represent respectively mean and SD response to irradiation for all samples. The black dot line corresponds to miRNAs with the same Cq in irradiated and non-irradiated samples. Colored dots represent significantly modulated miRNA (bilateral paired t-test p<0.05, n=3 or n=4). Empty dots represent control small RNAs RNU6-1 (triangle), SNORD44 (circle) and SNORD48 (square) response to ionizing irradiation.

We then focused on miRNA profiles after irradiation in proliferating and differentiated keratinocytes. After the high dose of 6 Gy, a global trend of repression was observed in proliferating keratinocytes whereas an opposite effect was observed in differentiated cells where most of the expressed miRNAs were induced after irradiation (Figure 2C and Figure 3 top and medium panel). After the very low dose, a slight increase of the miRNA expression profile was observed in proliferating keratinocytes (Figure 2C and Figure 3 bottom panel). These data revealed that the miRNA response follows a global trend that is highly dependent on the radiation dose and the differentiation status of irradiated cells.

**Figure 4:**
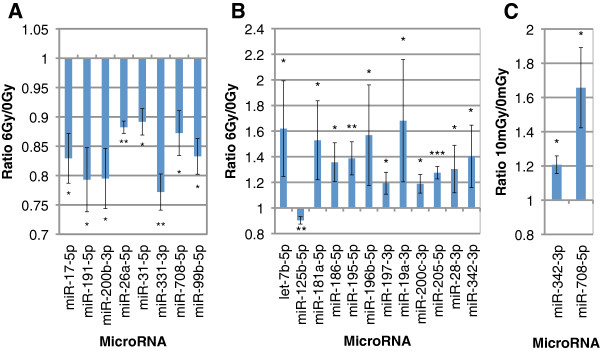
**MiRNAs significantly modulated by ionizing irradiation in human skin primary keratinocytes. A**. MiRNAs significantly modulated in proliferating keratinocytes exposed to 6 Gy (n=3 ; error bars show SD ; bilateral paired t-test : *p≤0.05, **p≤0.01) **B**. MiRNAs significantly modulated in differentiated keratinocytes exposed to 6 Gy (n=4 ; error bars show SD ; bilateral paired t-test : *p≤0.05, **p≤0.01, ***p≤0.001) **C**. MiRNAs significantly modulated in proliferating keratinocytes exposed to 10 mGy (n=3 ; error bars show SD ; bilateral paired t-test : *p≤0.05). The relative expression ratio (irradiated vs non-irradiated) is indicated.

Individual miRNAs significantly modulated after irradiation among the 3 or 4 independent cell samples were identified (colored dots in Figure 3). These miRNAs covered a wide range of expression from highly (Cq around 22 cycles) to weakly expressed (Cq around 31 cycles). After 6 Gy, 8 and 12 miRNAs were found to be significantly modulated in proliferating and differentiated cells respectively (Figure 4A and B). As expected with regards to the global response, miRNAs modulated in proliferating cells were all repressed whereas 11 out 12 miRNAs modulated in differentiated cells were induced. The miRNAs responding to the high dose of 6 Gy in proliferating cells were all different from those induced in differentiated keratinocytes. After 10 mGy, only 2 induced miRNAs were identified so far in proliferating keratinocytes (Figure 4C).

**Figure 5:**
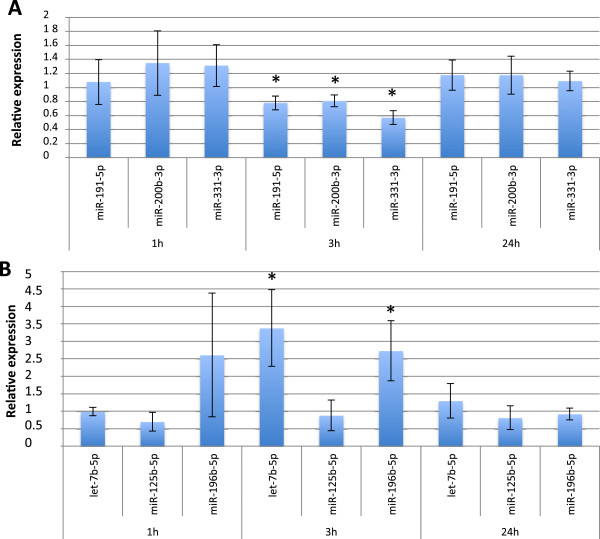
**Confirmation of TLDA data by individual quantitative real-time PCR (qRT-PCR).** Validation of TLDA data by qRT-PCR in irradiated (6Gy) vs non-irradiated proliferating (**A**) or differentiated (**B**) keratinocytes at 1, 3 and 24 hours post-IR. Values are mean fold changes +/− SD of independent experiments performed in triplicate on keratinocytes from 3 different donors (bilateral paired t-test : *p≤0.05).

To strengthen the validity of the data obtained from the large-scale TLDA analysis, we used individual QPCR to study the expression of 6 miRNAs in proliferating or differentiated keratinocytes among a time course between 1 and 24 hours post 6 Gy-irradiation. In proliferating keratinocytes (Figure 5A), we confirmed the down-regulation of the 3 tested miRNAs (miR-191-5p, miR-200b-3p and miR-331-3p) at 3 h whereas no modulation was detected at 1 or 24 h. In differentiated keratinocytes (Figure 5B), we also observed a significant response at 3 h for two of the 3 tested miRNAs: as observed after TLDA analysis (Figure 4B), let-7b-5p and miR-196b-5p were up-regulated 3 hours after irradiation. Globally, these data are in accordance with those obtained from the large-scale TLDA analysis. They also support the choice of the 3 hours time-point as a key moment in the miRNA response of irradiated keratinocytes.

### Towards mechanisms of the miRNA response

**Figure 6:**
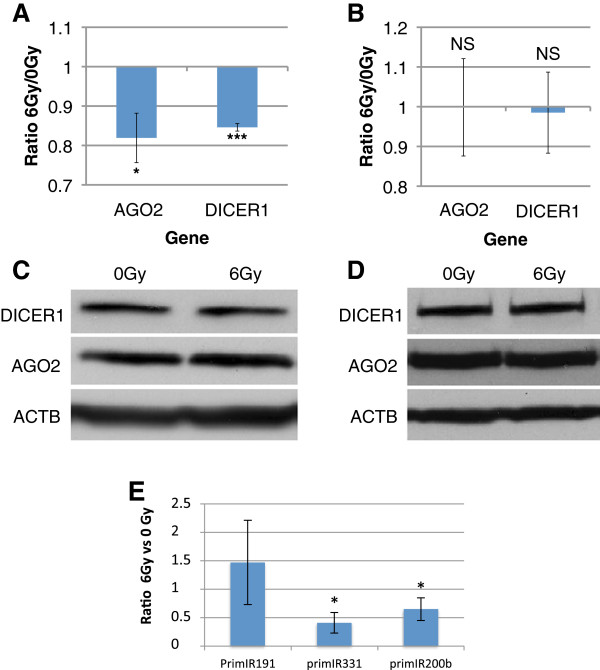
**Impact of ionizing radiation on the miRNA biogenesis pathway.** The radiation-response of AGO2 and DICER, that are involved in the late steps of the miRNA processing pathway, was assessed at the mRNA and protein levels. AGO2 and DICER1 mRNA expression 3 h after 6-Gy irradiation in proliferating (**A**) and differentiated keratinocytes (**B**) addressed by RT-QPCR (n=3; error bars represent SD; bilateral paired t-test : *p≤0.05, ***p≤0.001, NS: Non-significant). AGO2 and DICER1 protein level 3 h after 6-Gy irradiation in proliferating (**C**) and differentiated keratinocytes (**D**) addressed by immunoblotting. ACTB (Actin B) was used as loading control. (**E**) The expression of the primiR-191, primiR-200b and primiR-331 was assessed by quantitative qRT-PCR in proliferating keratinocytes 3 hours after 6 Gy-irradiation. Values are mean fold changes +/− SD of independent experiments performed in triplicate on keratinocytes from 3 different donors (bilateral paired t-test: *p≤0.05).

To go further into the mechanisms of this miRNA response after IR, we analyzed the effect of radiations on two proteins known to involved in the processing of miRNAs: DICER1 and AGO2 [18]. Indeed, DICER1 is an RNAse III endonuclease that acts in the endonucleolytic processing of miRNAs by cleaving their loop to release a duplex RNA stem [27]. This duplex RNA is incorporated into the RISC complex, a multi-protein complex that also contains the Argonaute proteins (AGO1 to AGO4 in humans). Therefore, we analyzed the mRNA response of DICER1 and AGO2 genes in proliferating and differentiated keratinocytes 3 hours after 6 Gy irradiation. In proliferating cells, we observed a faint but significant decrease of mRNA expression level for both DICER1 and AGO2 (Student *t*-test, *P* < 0.05 and < 10^-3^ respectively) (Figure 6A), whereas protein amount was not significantly different (Figure 6C). In differentiated cells, no modulation was detected either at RNA or protein level for both DICER1 and AGO2 (Figure 6B and D). These data suggest that the late steps of the miRNA-processing pathway, that require AGO2 and DICER, are not directly impacted by ionizing radiations. To have a look to the first step of miRNA biogenesis, we analyzed the relative expression of the primary transcripts corresponding to 3 repressed miRNAs in proliferating keratinocytes. A significant down-regulation was observed for primiR-331 and primiR-200b in 6 Gy-irradiated keratinocytes (Figure 6E). These results indicate that some IR-responding miRNAs are directly regulated at the transcriptional level. As miR-191-5p is down-regulated whereas the level of its precursor remains constant, those results suggest that the global modulation of the miRNAs expression that we detected after irradiation (see Figure 3) is the result of multiple and complementary levels of regulation within the miRNA biogenesis pathway.

### Functional relevance of IR-responding miRNAs

**Figure 7:**
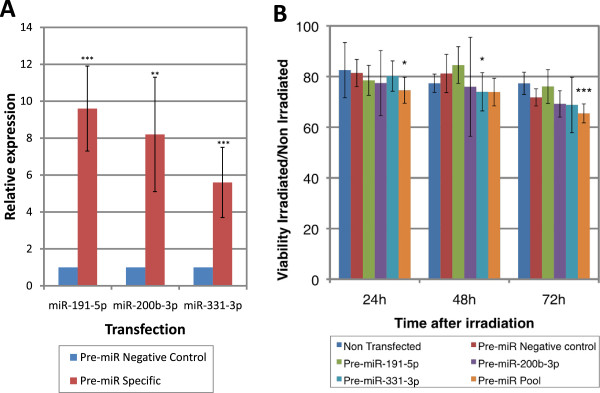
**Viability assays after modulation of microRNA expression in proliferating keratinocytes. A**. Proliferating keratinocytes were transfected with pre-miRNA for miR-191-5p or miR-200b-3p or miR-331-3p (pre-miR-specific) or with a Pre-miR negative control. MiRNA relative expression was assessed 48 h after transfection by RT-QPCR (bilateral paired t-test: *p≤0.05, **p≤0.01, ***p≤0.001). **B**. The relative 6Gy/0Gy viability (reported to the pre-miR negative control transfection) is indicated for each individual or combined (pool) pre-miR transfection at 24 h, 48 h and 72 h post-irradiation (n=4 ; error bars show SD ; bilateral paired t-test : *p≤0.05, ***p≤0.001).

After identification of radiation-responsive miRNAs, our aim was to test their individual or global impact on the survival of irradiated cells. We decided to focus first on proliferating cells that are more radio-sensitive (see Figure 1B). Because of their higher level of repression in irradiated proliferating cells, miR-191-5p, miR-200b-3p and miR-331-3p were selected for further analysis. These 3 miRNAs were individually over-expressed before irradiation by transfection of the corresponding pre-miRNA. In parallel, a pool of the 3 miRNAs was also over-expressed in proliferating keratinocytes prior to irradiation. The experimental conditions were adapted to ensure a moderate over-expression (5 to 10 times) of the miRNAs in transfected cells (Figure 7A). Over-expression of individual miRNAs had no effect on cell survival (except for miR-331-3p at 48 h) whereas a combination of the 3 miRNAs leads to a significant reduction of cell viability especially at 72 h post-irradiation (Figure 7B and Table 1). We also observed that some combined over-expression of two of the 3 miRNAs were also able to significantly reduced cell survival at 72 h (Additional File 2). These data suggest that the miRNAs that are modulated in irradiated cells plays a role in the cellular radiation response through a combined mode of action. This action is probably exerted through the regulation of downstream targets genes. To go further into these mechanisms, we searched for putative gene targets of miR-191-5p, miR-200b-3p and miR-331-3p. By crossing the results of five prediction programs (miRanda, miRDB, miRWalk, RNA22 and TargetScan), we identified hundreds of genes targets for each miRNAs. Among these genes, the 3 miRNAs share 83 targets. 783 genes are potentially regulated by at least 2 of the 3 miRNAs (see Venn diagram in Additional File 3 and the complete list of these targets in Additional File 4). A functional annotation of these 783 putative targets using GOrilla (Gene Ontology enrichment analysis and vizualization tool) revealed an enrichment of genes involved in the modulation of G-protein coupled receptor-signaling pathway through adenylate cyclase (Enrichment p-value = 9.8 10^-6^, FDR q-value = 3.9 10^-2^).

**Table 1:** P-values for viability assays after individual or pooled miRNA transfection

	**24 h**	**48 h**	**72 h**
**Pre-miR-191-5p**	0.5097	0.8005	0.4334
**Pre-mIR-200b-3p**	0.5139	0.7061	0.7757
**Pre-miR-331-3p**	0.7780	0.0199	0.6822
**Pre-miR Pool**	0.0137	0.0651	0.0008

The P-values of bilateral paired t-test used to assess the significance of the viability assays 24 h, 48 h or 72 h after irradiation are given.

We used the same strategy to explore the functional impact of the 6 Gy-responding miRNAs in differentiated cells. A combined silencing of the 3 more induced sequences (let7b-5p, miR-196b-5p and miR-19a-3p) by transfection of the corresponding antagomiR had no effect on cell survival at 72 h post-irradiation (data not shown).

## Discussion

In this work, we identified several miRNAs responding to IR in human keratinocytes. In proliferating cells exposed to 6 Gy, we identified 8 miRNAs that are repressed 3 hours after exposure. Among these 8 miRNAs, only miR-17-5p [28] and miR-31-5p [29] have been previously found to be deregulated by IR in other primary or cancer cells. Moreover, miR-31-5p has been shown to repress the expression of several genes involved in DNA repair in adenocarcinoma cell lines [29]. Its down-regulation in proliferating keratinocytes might contribute to increase the DNA repair efficiency after irradiation. MiR-99b-5p was also described as playing a role in DNA repair. The miR-99 family, including miR-99b-5p, regulates the DNA-damage response in breast and prostate cancer cells by targeting the chromatin remodeling factor SNF2H [30]. When the miR-99 cluster is over-expressed in irradiated cells, the rate and the overall efficiency of repair by both NHEJ and homologous recombination are reduced [30]. In our study, the down-regulation of miR-99b-5p could contribute to an activation of double-strand breaks repair in irradiated keratinocytes. Irradiation of proliferating keratinocytes leads to a cell cycle arrest that is requested for DNA repair [31]. Some of the miRNAs repressed by IR in proliferating keratinocytes could be involved in this growth arrest: it is the case of miR-17-5p and miR-191-5p that are over-expressed in cancer cells where they contribute to activate cell proliferation [32,33]. In differentiated keratinocytes exposed to 6 Gy, we identified 12 responding miRNAs, all except miR-125b-5p were induced in irradiated cells. Among these 12 miRNAs only let-7b-5p has been previously described as being modulated by ionizing radiations in primary or cancer cells [34]. This suggests that the miRNA response elicited by IR in human keratinocytes is very specific to this cell type and also dependent to the differentiation status of irradiated keratinocytes. In a recent study, Zhou et al. investigated the miRNA profile of UVB-irradiated normal human keratinocytes. Among the 44 miRNAs that they found modulated by UVB, only 4 (miR-31-5p, let-7b-5p, miR-125b-5p and miR-186-5p) also responded to IR in our study [35]. This could indicate that UVB and IR provoke specific effects in exposed keratinocytes that in turn elicit a stress-specific miRNA response. It has been previously observed that genotoxic stresses, such as UV or IR, induce a very specific response in differentiated keratinocytes including induction of terminal differentiation [36,37]: among the 12 miRNAs that we found induced after irradiation in differentiated keratinocytes, 5 of them (miR-125b-5p, let-7b-5p, miR-181a-5p, miR-195-5p and miR-342-3p) have been previously observed as being also induced during the differentiation process of human keratinocytes [38]. These 5 radiation-responding miRNAs could be directly involved into the modification of the differentiation program that is elicited by gamma irradiation in differentiated keratinocytes.

The main question raised by our data is the biological significance of this miRNA response. We have re-expressed 3 down-regulated miRNAs in irradiated proliferating keratinocytes. We observed that a combined over-expression of these miRNAs reduced cell viability 72 hours post-IR. These results indicate that the down-regulation of these miRNAs is important for cell survival after IR. This could also evocate a coordinated function of these miRNAs in the cellular radiation response probably through the regulation of common genes targets. We identified more than 780 potential targets in common between at least two of these 3 responding miRNAs. Functional annotation of this list revealed an over-representation of genes involved in the adenylate cyclase G-protein coupled receptor-signaling pathway. The cAMP signaling pathway has been shown to modulate DNA-damaging agents induced apoptosis and DNA repair activity [39,40]. This pathway could be one of the mechanisms downstream to ionizing-radiation-responding miRNAs and necessary for the survival of irradiated cells. In differentiated keratinocytes, the silencing of the 3 more responding miRNAs had no effects on immediate cell viability: this suggests that the miRNAs responding to IR in differentiated keratinocytes are not directly involved in immediate cell survival. Since these cells are already engaged in a differentiation process that will finally lead to cornification, a specialized form of programmed cell death, the activation of the differentiation process might be a way to eliminate damaged cells. As discussed above, this hypothesis was supported by the fact that, in differentiated keratinocytes, several miRNAs that we found induced by IR are also markers of epidermal differentiation.

Several studies have been conducted to study the effects of low doses ranging from 50 mGy to 100 mGy on miRNAs expression in various cellular models including fibroblasts [17,41], blood cells [42] or thyroid cells [43]. However, to our knowledge, the present study is the first to address the miRNA response of human cells after a very low dose of 10 mGy. This dose is relevant in the context of medical imaging procedures where 20% of the patients can be potentially exposed to radiation between 3 and 20 mGy, especially after abdominal compute tomography [41]. Contrary to the high dose of 6 Gy where all the responding miRNAs are down-regulated in proliferating keratinocytes, the very low dose of 10 mGy leads to a particular response with only two up-regulated miRNAs: miR-342-3p and miR-708-5p. As previously observed for mRNA [21], a specific miRNA response is then detected in human keratinocytes after the very low dose of 10 mGy. This might reflect the emerging concept that the dose response is not linear and that very low doses of IR induce specific cellular mechanisms in irradiated cells [44]. Further experiments will be necessary to precise how miR-342-3p and miR-708-5p might be involved in this particular response.

## Conclusion

Our results suggest that in human keratinocytes the expression of miRNAs in response to ionizing radiation is highly dependent to the differentiation status of irradiated cells. It is also clear from our study that the miRNAs response is dose dependent and that a very low dose of 10 mGy is able to modulate some miRNAs. Since deregulation of specific miRNAs has the consequence of changing cell survival after irradiation, this might represents a possible strategy to modulate cellular sensitivity to anti-cancer treatments.

## Methods

### Ethical considerations

Infant foreskins were collected according to the Declaration of Helsinki Principles. A written informed consent was obtained from infants’ parents according to french bioethical law of 2004 (loi 94–654 du 29 juillet 1994).

### Cell culture

Foreskins from healthy boys were conserved at 4°C in DMEM supplemented by 10% FBS and penicillin/streptomycin till keratinocytes preparation. Keratinocyte were isolated from epidermis using overnight 4°C dispase/trypsin digestion. Cells were seeded on BioCoat™ Collagen I cellware (Becton-Dickinson) in KGM2 medium (PromoCell) supplemented with 100 μg.ml^-1^ antimicrobial Primocin™ (InvivoGen). Subconfluent cultures were passed using 0.05% trypsin/EDTA (GIBCO) and reseeded three-time up for studies in KGM2 medium. Keratinocytes differentiation was induced at confluence adding CaCl_2_ to KGM2 medium, raising calcium concentration from 0.06 to 1.8 mM.

### Samples irradiation

Proliferating and three-days differentiated keratinocytes were 6 Gy-irradiated using a Cs^137^ source (IBL637 Cisbio international) with a dose-rate of 0.62 Gy.min^-1^ calculated on radioactive decay. Proliferating keratinocytes were 10 mGy-irradiated at the French army health service research facility (CRSSA) on IRDI 4000 (Alstom) with a dose-rate of 21.68 mGy.min^-1^ calculated on radioactive decay. Samples irradiation was performed in biological triplicate or tetraplicate.

### RNA extraction and quality evaluation

Total RNA was prepared using mirVana™ miRNA Isolation Kit (Applied Biosystems) according to the manufacturer’s instructions and conserved at −80°C till use. RNA purity and concentration were assayed using a NanoDrop 2000 Spectrophotometer (Thermo Scientific). All samples presented A^260^/A^280^ ratios > 2 and A^260^/A^230^ ratios > 1.9 in DEPC-treated water. RNA integrity was checked using a 2100 Bioanalyser (Agilent). All samples harbored correct 18S/28S profile and a RIN factor between 8.9 and 10.

### MiRNA profiling and analysis

RT-qPCR experiments were performed by profileXpert platform (Université Claude-Bernard Lyon 1). The quantification of the 667 human miRNAs was performed using TaqMan® Array Human MicroRNA Cards A and B v2.0 (4400239, Applied biosystems) according to manufacturer instructions. Briefly, miRNAs were retrotranscribed by Megaplex™ RT Primers kit. RT primers are based on miRBase v14. Complementary DNA (cDNA) were plated on cards and PCR were performed on 7900HT Fast Real-Time PCR System (Applied Biosystems). Raw Cq (quantification Cycle) values were calculated using the RQ Manager version 1.2 applying automatic baseline settings and a threshold of 0.15. Plant ath-miR159a served as negative control. Cq threshold of 32 was calculated using miRNA duplicate of card B and snRNA tetraplicate repeatability, confirmed by literature [25]. Only miRNA with Cq below the threshold in all condition by state and irradiation dose were sorted. Data analysis was performed using JMP 5.0.1.2 software (SAS Institute). For miRNA targets prediction, we used five different miRNA target prediction tools: miRanda, miRDB, miRWalk, RNA22 and TargetScan. From all predictions, we considered miRNA-transcript relationships that were predicted by a minimum of two different tools. In addition, we performed a Gene Ontology term enrichment analysis using the GOrilla application [45]. To correct for multiple testing, we used the Benjamini-Hochberg procedure and considered an association significant if the P value was less than 0.05.

### Reverse transcription and quantitative PCR for mRNA

Contaminating DNA was removed from 500 ng of RNA using DNaseI (EN0521, Fermentas) at 0.1U.μl^-1^ in 10 μl at 37°C during 30 min. DNA-free RNA was reverse transcribed using SuperScript™ II Reverse Transcriptase (18064–014, Invitrogen) with 17 ng of oligo-d(T) (Amersham Pharmacia Biotech Inc.) and 5 ng of random hexamers d(N)_6_ (N8080127, Invitrogen). RT products were diluted to an equivalent RNA concentration of 10 ng per well. Samples and standards were manually plated in duplicate on 96 wells polypropylene plates (410088, Agilent Technologies). Non-template controls were performed for each assay in all experiment and displayed not detectable or significant amplification. Quantitative PCR (qPCR) were performed using ABsolute™ Blue QPCR SYBR® Green ROX Mix (AB-4163/A, ThermoScientific) with Mx3000 system (Stratagene). Primer design was made using PrimerBlast interface (http://www.ncbi.nlm.nih.gov/tools/primer-blast/). PCR reactions were initiated by the activation of Taq DNA polymerase at 95°C for 15 min, followed by 45 three-step amplification cycles consisting of 15 s denaturation at 95°C, 30 s annealing at 58-60°C and 30 s extension at 72°C. After the amplification, a dissociation stage was run to generate a melting curve for verification of amplification product specificity, which was also confirmed on agarose gels. ROX normalized and automatic baseline corrected quantification cycles (Cq) were collected using MxPro software (version 3.00b, Stratagene). Amplification efficiencies were calculated using linear regression of cDNA pool dilution and between 90% and 105%. Standard minus square correlations were above 0.995. Relative quantification was calculated using standard curve efficiency-corrected comparative quantification [46] using Microsoft® Excel® Mac 2008. 18S served as differentiation-related reference gene. Ubiquitin c (UBC) served as ionizing irradiation-related reference gene as one of the most invariant gene in this stress [21]. The nucleotide sequence and the amplicon length for of all the primers used in this study are given in the Additional file 5. The MIQE checklist [47] of our QPCR experiments is given is the Additional file 6.

### Reverse transcription and quantitative PCR for miRNA and primiRNAs

PrimiRNA were reverse transcripted from 500 ng of total RNA using the PrimeScript™ RT reagent kit (RR037A, Takara) according to the manufacturer instructions. Mature miRNAs from 100 μg of total RNA were retrotranscribed using TaqMan® MiRNA Reverse Transcription Kit (4366596, Applied Biosystems™) according to the manufacturer protocol. QPCR were performed in duplicate. Non-template controls and No-RT controls were performed for each assay in all experiment and display not detectable amplification. Quantitative PCR were performed using TaqMan® primiRNA or miRNA Assays and TaqMan® Universal PCR Master Mix (Applied Biosystems). Relative quantification was calculated using 2ΔΔ Cq quantification method [48]. The UBC gene, that we have previously shown to be invariant after irradiation in human keratinocytes [21], was used as a reference to normalize QPCR for primiRNA quantification. MiR-125b-5p and miR-17-5p were used as reference to normalize individual QPCR after irradiation on proliferating and differentiated keratinocytes respectively. RNU6-1 served as reference for QPCR after pre-miR transfection assays.

### Ionizing irradiation viability assay

Viability was performed with resazurin/resorufin-based alamarBlue® cell viability assay (Life Technologies™). Keratinocytes were seeded at 8000 cell per cm^2^. The day after, cells were transfected during 6 h with 50pM pre-miR-191-5p, 50pM pre-miR-331-3p or 500fM pre-miR-200b-3p, individually or pooled, in tetraplicate using HiPerfect Transfection Reagent (301705, Qiagen). Days after transfection, 10% v/v alamarBlue was added to culture medium and fluorescence was read in technical duplicate 4 h after addition using Xenius instrument (Safas).

### Western blot

Protein extraction was performed using RadioImmunoPrecipitation Assay medium (RIPA, [49]). Protein concentrations were measured by Bradford method using Bio-Rad Protein Assay (500–0006, Bio-Rad). Electrophoresis was performed with 20 ng of protein. Western blot were performed as described previously [4]. Results were obtained from four independent donors. EIF2C2 (ab57113) and DICER1 (ab14601) antibodies were purchased from abcam® and used after 1/200 and 1/1000 dilution respectively. ACTB, clone C4 (MAB1501) antibody was purchased from Merck Millipore and used after 1/2000 dilution.

## Abbreviations

IR: Ionizing radiations; miRNA: microRNA; HPK: Human Primary Keratinocytes; RT-QPCR: Reverse Transcriptase Quantitative Polymerase Chain reaction; TLDA: Taqman quantitative PCR Low Density Array; Cq: Quantification Cycle; SD: Standard Deviation; PCNA: Proliferating Cell Nuclear Antigen

## Competing interests

The authors declare that they have no competing interests.

## Authors’ contribution

NJT performed experiments, analyzed data and wrote the manuscript. JV performed statistical analysis and wrote the manuscript. OG designed experiments and analyzed data. JL designed experiments, analyzed data and wrote the manuscript. All authors read and approved the final manuscript.

## Supplementary Material

Additional file 1 **Full data of miRNAs analyzed in our study.** The Cq and relative expression for all analyzed miRNAs are given for the 3 conditions (Proliferating cells 6 Gy, Differentiated Cells 6 Gy, Proliferating cells 10 mGy). Click here for file

Additional file 2 **Viability assays after combined up-regulation of two miRNAs in proliferating keratinocytes.** The relative 6Gy/0Gy viability (reported to the pre-miR negative control transfection) is indicated for each combination of two pre-miRNAs transfection at 24 h, 48 h and 72 h post-irradiation (n=4 ; error bars show SD ; bilateral paired t-test : ***p≤0.001). Click here for file

Additional file 3 Venn diagram showing the predicted targets in common between the 3 miRNAs miR-191-5p, miR200b-3p and miR-331-3p. Click here for file

Additional file 4 List of the predicted targets for the microRNAs miR-191-5p, miR200b-3p and miR-331-3p and list of the targets in common between these 3 miRNAs. Click here for file

Additional file 5 **Primers Table.** Primers specificity was assayed by PrimerBLAST or Primer3. Primers were designed to amplify all mRNA variants if exist. Primers were purified by SePOP. Click here for file

Additional file 6 Minimum information for publication of quantitative real-time PCR experiments (MIQE) checklist. Click here for file
