# Supplementary figures and images for: Differential miRNA expression profiles in proliferating or differentiated keratinocytes in response to gamma irradiation
Source: BMC Genomics. 2013 Mar 16;14:184. doi: 10.1186/1471-2164-14-184 (PMC3610249; doi:10.1186/1471-2164-14-184)

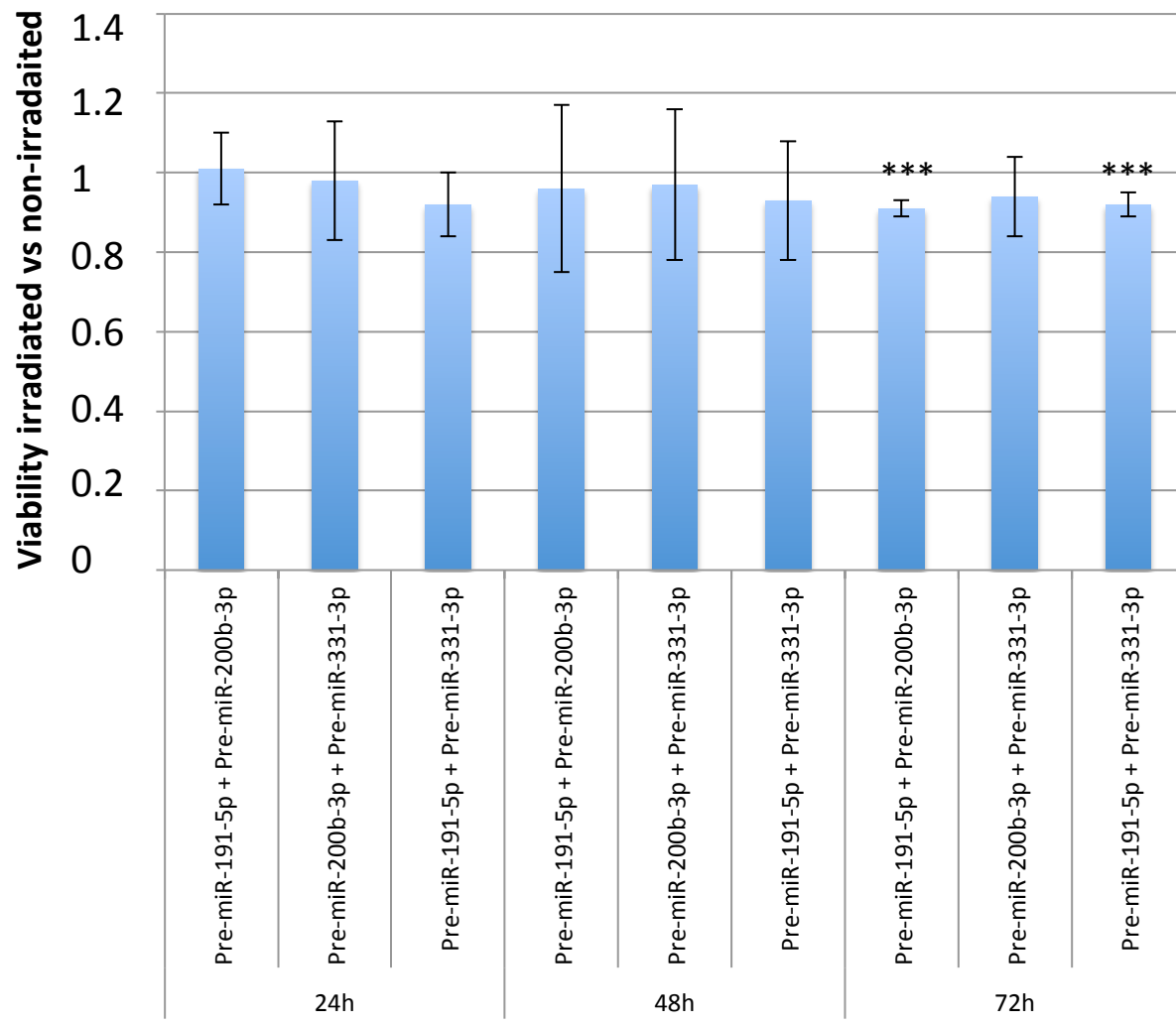

Supplement: Additional file 2 — Viability assays after combined up-regulation of two miRNAs in proliferating keratinocytes. The relative 6Gy/0Gy viability (reported to the pre-miR negative control transfection) is indicated for each combination of two pre-miRNAs transfection at 24 h, 48 h and 72 h post-irradiation (n=4 ; error bars show SD ; bilateral paired t-test : ***p≤0.001). [file 1471-2164-14-184-S2.pdf]

miR-200b-3p

miR-331-3p

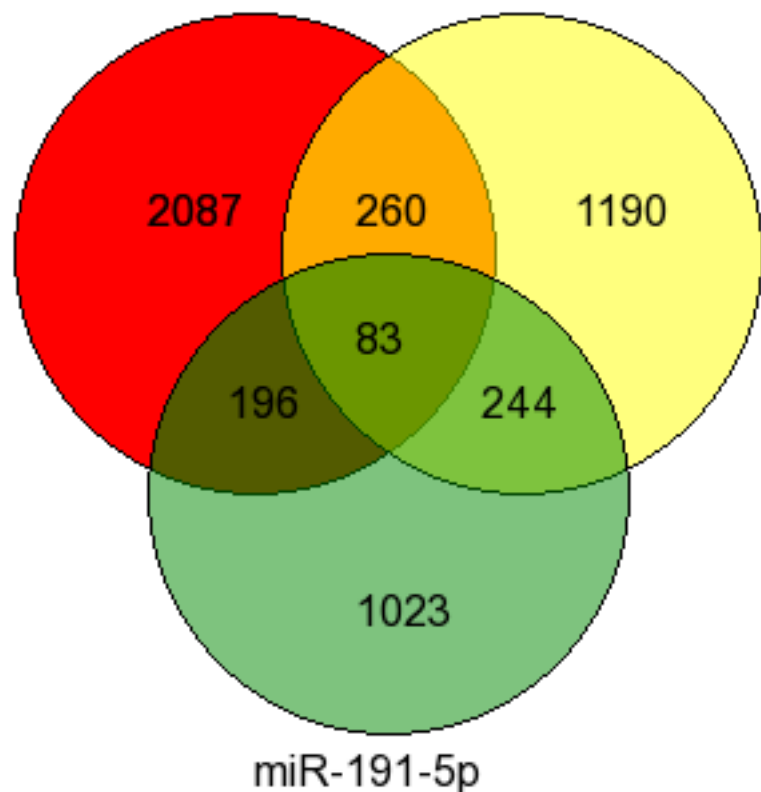

Supplement: Additional file 3 — Venn diagram showing the predicted targets in common between the 3 miRNAs miR-191-5p, miR200b-3p and miR-331-3p. [file 1471-2164-14-184-S3.pdf]
