# Supplementary material for: Differential miRNA expression profiles in proliferating or differentiated keratinocytes in response to gamma irradiation
Source: BMC Genomics. 2013 Mar 16;14:184. doi: 10.1186/1471-2164-14-184 (PMC3610249; doi:10.1186/1471-2164-14-184)
Supplement: Additional file 5 — Primers Table. Primers specificity was assayed by PrimerBLAST or Primer3. Primers were designed to amplify all mRNA variants if exist. Primers were purified by SePOP. [file 1471-2164-14-184-S5.docx]

| Gene | Gene ID | Amplicon Lenght | Primer | Primer Sequence |
| --- | --- | --- | --- | --- |
| UBC | 7316 | 176 | Reverse | 5’ CGCCTGTTCCGCTCTCTGGAAA |
|  |  |  | Forward | 5’ CACGTCAGACGAAGGGCGCAG |
| DICER1 | 23405 | 194 | Reverse | 5’ CTACTTCCACAGTGACTCTG |
|  |  |  | Forward | 5’ CATGGATAGTGGGATGTCAC |
| EIF2C2 | 27161 | 139 | Reverse | 5’ TGGCTGTGCCTTGTAAAACGCT |
|  |  |  | Forward | 5’ CGCGTCCGAAGGCTGCTCTA |
| 18S | 100008588 | 125 | Reverse | 5’ CGATGCGGCGGCGTTATT |
|  |  |  | Forward | 5’ CCTGGTGGTGCCCTTCCGT |
| KRT14 | 3861 | 117 | Reverse | 5’ ATCCTCCCCCAAAGCCACTAC |
|  |  |  | Forward | 5’ GCCTGTCTGTCTCATCCTCCC |
| PCNA | 5111 | 113 | Reverse | 5’ GACTTTCCTCCTTCCCGCC |
|  |  |  | Forward | 5’ CCTTGAGTGCCTCCAACACC |
| KRT1 | 3848 | 146 | Reverse | 5’ TGGTAGAGTGCTGTAAGGAAATCAATT |
|  |  |  | Forward | 5’ GATGAAATCAACAAGCGGACAA |
| IVL | 3713 | 126 | Reverse | 5’ GCAGTCATGTGCTTTTCCTCTTG |
|  |  |  | Forward | 5’ CTGCGGAGGTGGTTCCTCT |
